# Adolescents’ Self-Regulation of Social Media Use During the Beginning of the COVID-19 Pandemic: An Idiographic Approach

**DOI:** 10.1007/s41347-024-00465-z

**Published:** 2024-12-17

**Authors:** Melissa J. Dreier, Carissa A. Low, Jennifer Fedor, Krina C. Durica, Jessica L. Hamilton

**Affiliations:** 1 Department of Psychology, Rutgers University, 50 Joyce Kilmer Road, Piscataway, NJ 08854 USA; 2 Department of Medicine, Division Hematology/Oncology, University of Pittsburgh, Pittsburgh, PA USA

**Keywords:** Adolescent, Social media, Mood, Affect, Mental health, Idiographic

## Abstract

Adolescent social media serves a broad range of functions, which may be helpful for some and harmful for others. During the COVID-19 lockdown, social media evolved considerably, occupying an even more central role in adolescents’ lives. This study leverages a new approach to measuring social media use behaviors—passive smartphone sensing. Specifically, we aimed to test if and how adolescents self-regulate their social media use in response to how they feel during and after use. This study followed 19 adolescents for 1 month. Participants completed baseline measures, assessing demographic and clinical characteristics. We used passive smartphone sensing to measure objective social media use behaviors (“screen time” and checking) for a 1-month period. Adolescents also completed daily diary questions on their mood. Analyses took an idiographic (*n* = 1) approach. Dynamic structural equation models tested daily and next-day relationships between social media use behaviors and mood for each adolescent. Most adolescents (*n* = 13 of 19) did not self-regulate their social media use in relation to their mood. Most importantly, they did not use it less when they felt more negative mood during use. That said, some adolescents (*n* = 6) did alter their social media use behaviors depending on their mood. Each adolescent’s pattern of social media use and mood was also qualitatively interpreted within their context of demographic (e.g., experience of holding a minoritized identity) and clinical characteristics (e.g., history of suicidal thoughts and behaviors). These results highlight the next steps for possible intervention points to help adolescents adjust their use patterns to maximize mental health benefits while minimizing possible harm. Findings also begin to develop a template for applying social media use recommendations, while centering the experiences of individual adolescents.

## Introduction

The adolescent mental health crisis has been receiving increasing attention in popular media and news outlets, and many journalists are citing social media—or online platforms that allow for both synchronous and asynchronous social interaction (Carr & Hayes, 2015; Charmaraman et al., 2022a)—as the cause (e.g., Hughes, 2022; McCluskey, 2021). Indeed, as mental health concerns—like suicidal thoughts and behaviors—have been increasing (National Center for Health Statistics, 2021), so has the percentage of teenagers who report being online “almost constantly” (Anderson et al., 2023; Vogels et al., 2022). Yet, research to date examining the association between social media and mental health outcomes indicates positive, negative, *and* null relationships for adolescents depending on the individual (Berryman et al., 2018; Hamilton et al., 2021a, 2021b, 2021c; Montag et al., 2024; Nesi et al., 2021). The COVID-19 pandemic also reshaped many adolescents’ relationships with social media, such that adolescents tended to use it more frequently and used more video-based platforms, like TikTok, which more closely mimic the real world than photo-based platforms (Charmaraman et al., 2022b; Hamilton et al., 2023; Hamilton, Nesi et al., 2021). Similar to research before the pandemic, meta-analytic research finds that there was a small association between adolescent social media use and well-being during the pandemic (Marciano et al., 2022). However, different studies find different effects, highlighting likely individual variability (Marciano et al., 2022).

### Starting Small: Links Between Social Media Use and Mood

To understand the big-picture links between social media and adolescent mental health, it is necessary to first zoom in and understand how social media affects adolescents’ mood on a short timescale or more proximally. Understanding this link may uncover “adaptive” and “maladaptive” patterns of social media use on a more proximal timescale than broad associations between social media and mental health outcomes allow. Within-person increases in negative experiences on social media are associated with sustained negative mood (Boyd et al., Under review; Hamilton et al., 2021a, 2021b, 2021c). A higher frequency of phone use in general (e.g., calls and texts) is associated with symptoms of anxiety and depression, even within a single day (Rodman et al., 2021). On the flip side, adolescents tend to experience a boost in positive mood when they use social media (Minich & Moreno, 2024) and they check it more frequently when they feel more positive mood than usual while using it (Dreier et al., 2024). These proximal links point to the fact that social media could “hit a nerve” for many adolescents, possibly causing more mood lability than in-person interactions. That said, most research to date on this topic is cross-sectional and leaves open questions about how different adolescents use social media in response to their mood and what constitutes “adaptive” versus “maladaptive” patterns of use.

### Centering Individual Adolescents in Broad Recommendations

It is additionally important to situate individual adolescents’ characteristics within our broad understanding of adolescent social media use habits. Adolescents with minoritized identities experience unique benefits and harms on social media. Among youth of color, experiences of online discrimination, including vicarious discrimination, are associated with mental health diagnoses, like anxiety and depression (Tao & Fisher, 2022; Thomas et al., 2023). On the other hand, LGBTQIA + adolescents often seek supportive communities on social media (Escobar-Viera et al., 2022; Gordon et al., 2023). Adolescents’ prior mental health history may also be important to consider. Social media may expose adolescents to harmful content, like pro-suicide and pro-eating disorder communities (Fitzsimmons-Craft et al., 2020; Minkkinen et al., 2017). However, for adolescents with recent suicide attempts, *more* social media use is associated with a *lower* risk for a subsequent suicide plan (Hamilton et al., 2021a, 2021b, 2021c). When crafting recommendations for adolescent social media use and mental health, it is important to consider how they may apply to individual adolescents with unique sets of identities and clinical characteristics.

### Novel Methods to Investigate Gaps in the Literature

In recent years, clinical psychology researchers have begun to apply idiographic methods to help the field realize its initial purpose: to use science to help individuals experiencing unique sets of behavioral patterns and symptoms (Molenaar, 2004). Recently, idiographic methods have been applied to understanding social media use, finding high between-person variability in the psychosocial effects of social media use and social media use experiences (Beyens et al., 2020, 2021; Rodriguez et al., 2022; Valkenburg et al., 2021, 2022). However, to date, this work has focused on self-reported social media experiences (e.g., what adolescents saw on social media) and behaviors (e.g., asking adolescents whether they browsed or posted on social media that day).

Measuring social media use objectively has been difficult to do until recently. Self-reported social media use is plagued by recall bias and is only moderately associated with objective measures of social media use (Parry et al., 2021; Sewall et al., 2022). There is also a notable dearth of studies that report on social media checking frequency (i.e., how often participants check social media, irrespective of the time they spend checking it). Social media checking may be more closely linked to adolescents’ mood lability than “screen time” is (Dreier et al., 2024) and more closely maps on to feedback-oriented social media behaviors (Thorisdottir et al., 2019).

Using this information in an idiographic framework, psychologists may move toward understanding a diverse array of ways in which social media use may interplay with adolescents’ mood. This could, one day, pave the way for evidence-based clinical guidelines on adolescent social media use that are not one-size-fits-all, but that could be tailored to individual adolescents and their unique habits/experiences.

### The Current Study

The current study used idiographic methods to understand how adolescents self-regulate their social media use, by examining the myriad of ways in which adolescents use social media in relation to their mood. This study explores person-specific patterns of social media use and mood on a daily level. This study also focuses on a sample of adolescents who completed data collection during the earlier months of the COVID-19 pandemic, shedding light on potentially important shifts in adolescent social media use during this time that may have ripple effects today. Findings from this study begin to develop a template for conceptualizing the ways in which social media use may uniquely affect individual adolescents, while still allowing for broader, empirically based conclusions that could inform public health recommendations. Specifically, this study answered three questions:

- Research Question (RQ1): How are social media “screen time” and social media checking associated with positive and negative mood during social media use on a proximal scale, over time, for individual adolescents?

To test this question, we used dynamic structural equation modeling (DSEM), an approach that allows researchers to construct structural equation models using repeated measures data and to extract both between-person and participant-level and within-person results (Asparouhov et al., 2018). Based on prior exploratory work on this topic (Dreier et al., 2024), we hypothesized that some adolescents will follow a “maladaptive” pattern, whereby they use or check social media more when experiencing a greater negative mood while using it. On the other hand, we hypothesized that other adolescents will exemplify an “adaptive” pattern of use, whereby they use or check social media more when they feel positively, but will reduce use when they feel negatively during use.

- Research Question 2 (RQ2): If and how do adolescents self-regulate their social media use (i.e., use or check it more or less) in relation to the general negative mood on the same day and following day?

To test this question, we also applied DSEM. We hypothesized that some adolescents will show an “adaptive” pattern of use, whereby, if social media tends to lead to a higher negative mood later in the day, they use it less the next day. We also predicted that other participants would show a “maladaptive” pattern, such that, even when the use of social media is associated with negative mood later in the day, they continue to use and/or check it at a similar or greater frequency the following day.

- Exploratory Research Question 3 (RQ3): What demographic and clinical characteristics are qualitatively associated with different social media use habits?

After classifying different patterns of “adaptive” and “maladaptive” social media use via RQ1 and RQ2, we qualitatively examined what characteristics are associated with different patterns of use. Though this research question is exploratory given limitations in power, we hypothesized that participants who engage in more “maladaptive” social media use may be experiencing symptoms of mental health problems (e.g., anxiety, depression, suicidal thoughts and behaviors), developmental transition (i.e., older adolescents), and potential exposure to systemic/structural inequities (e.g., financial insecurity, racism, homophobia). Although findings from RQ3 are neither empirical nor causal, they provide a starting template for beginning to assess different social media patterns in the context of developmental and systemic stressors.

## Method

### Data Collection

This study used data collected from the Social Media and Sleep Health (SMASH) study, which aimed to test real-time relationships between adolescent social media use, sleep, and mental health. Importantly, these data were collected in 2020, during the early months (April–November) of the COVID-19 pandemic. This study used passive smartphone sensing, actigraphy (via smart watches), and twice-daily surveys to investigate the relationships between social media use, sleep patterns, and suicidal thoughts and behaviors. As a part of data collection, participants agreed to install a passive sensing application on their smartphone that captured application usage, from which social media use (time spent using and number of times checked) could be derived. Participants also completed baseline questionnaires and twice-daily surveys every day for 1 month. This study was approved by the University Institutional Review Board. Participants under 18 years of age provided assent to participant and their parents provided informed consent. Participants who were 18 years old provided informed consent to participate.

### Study Sample

Adolescent participants were recruited through the Rutgers University online registry screening portal. Of the 21 adolescent participants who completed the SMASH study, a total of 19 had usable social media data. One excluded participant did not comply with passive sensing procedures and the other had parental controls that prevented capture of social media use data. To be included in the study, participants needed to be 13–18 years old, enrolled in a United States high school (9th through 12th grade), and speak English fluently. To participate in the social media passive sensing portion of the study, participants needed to use an Android phone, because the passive sensing software used in the study could not capture app use data from iPhone/iOS. Adolescents participated in the study for about one month, *M* (SD) = 31 (5.6) days. Table 1 presents demographic information on the 19 included study participants.

**Table 1:** Sample demographics for the 19 adolescents in the SMASH study

	*N* (%)
Age, *M* (SD)	15.84 (1.01)
Race	
White	15 (78.95%)
Black/African American	2 (10.53%)
More than one race	2 (10.53%)
Ethnicity	
Hispanic/Latine	0 (0%)
Non-Hispanic/Latine	19 (100%)
Sex	
Male	13 (68.42%)
Female	6 (31.58%)
Gender	
Boys	11 (57.89%)
Girls	7 (36.84%)
Non-binary/third gender	1 (5.26%)
Transgender	2 (10.53%)
Sexual orientation	
Heterosexual/straight	14 (73.68%)
Bisexual	3 (15.79%)
Queer	1 (5.26%)
Bi-curious	1 (5.26%)
SES—Society, *M* (SD; range)	6.37 (1.5; 4–9)
SES—School, *M* (SD; range)	6.53 (1.74; 4–10)
PDS, *M* (SD; range)	3.34 (0.35; 2.5–3.833)
MFQ, *M* (SD; range)	10.26 (11.61; 0–50)
MASC, *M* (SD; range)	41.58 (19.32; 4–85)
Suicidal ideation	6 (31.5%)
Suicide attempts	3 (15.8%)
Nonsuicidal self-injury	1 (5.26%)

Latine, a gender-neutral term for Latino/Latina; for both SES measures, 1 = much lower SES relative to these groups (society or school), and 10 = much higher SES relative to these groups

*M* mean, *SD* standard deviation, *SES—School* socioeconomic status relative to society at large, *SES—School* socioeconomic status relatives to others in the same school, *PDS* Pubertal Development Score, *MFQ* Mood and Feelings Questionnaire (Depression), *MASC* Multidimensional Anxiety Scale for Children

### Measures

Table 2 presents a summary of measures and constructs in the current study, described in further detail below.

**Table 2:** Summary of study measures and constructs

Construct	Measure/method	Citation	Baseline survey	Intensive monitoring
Social media use	AWARE passive smartphone sensing	(*AWARE – Open-Source Context Instrumentation Framework For Everyone*, 2014)		X
Mood during social media use	Visual analog scale: “How positive did you feel when using social media today?” and “How negative did you feel when using social media today?” Participants answered each question on a scale of 0 (no positive/negative feelings) to 100 (extreme positive/negative feelings)	N/A		X
Daily negative mood	Visual analog scale: “How sad/down would you rate your mood today?” on a scale of 0 (not at all sad/down) to 100 (extremely sad/down)	N/A		X
Demographics (age, race, ethnicity, sex, gender, sexual orientation)	Standard demographic questions	N/A	X	
Socioeconomic status	MacArthur Subjective Social Status Scale	(Goodman et al., 2001)	X	
Pubertal status	Pubertal Development Scale	(Petersen et al., 1988)	X	
Depression	Mood & Feelings Questionnaire	(Angold & Costello, 1987)	X	
Anxiety	Multidimensional Anxiety Scale for Children	(March et al., 1997)	X	
Suicidal thoughts and behaviors	Columbia Suicide Severity Rating Scale—Screening Version	(Posner et al., 2011)	X	

#### Core Study Variables (RQ1 and RQ2)

##### Social Media Use

Social media use was captured throughout the study using the AWARE framework (*AWARE – Open-Source Context Instrumentation Framework For Everyone*, 2014; Ferreira et al., 2015). This is an open-source platform whereby scientists can work with research participants directly to collect phone use data. Participants download the AWARE application on their phones. While this application is running in the background, researchers can access data on application usage in the foreground (ensuring it reflects active application use) to derive (1) how much time participants spend on each app on their phone and (2) how often they check each app over the study period. For each day participants were in the study, de-identified data from AWARE was uploaded to a cloud-based server.

Although AWARE collects data from a variety of phone sensors (e.g., app usage, light, movement), this study focuses on the application use sensor, which extracts the number of seconds per day that each application was used in the foreground (i.e., this excludes having an app open in the background on one’s phone) and how often adolescents open (i.e., check) each application. Collection of these data is near-continuous, as long as the AWARE application is running properly.

##### Mood During Social Media Use

To measure positive and negative mood *during* social media use, the study used daily evening surveys, which asked participants, “How positive did you feel when using social media today?” and “How negative did you feel when using social media today?” Participants answered each question on a scale of 0 (no positive/negative feelings) to 100 (extreme positive/negative feelings). Visual analog scale (VAS) response options are commonly used in psychology research to measure emotional experience (Hall et al., 2021), and this item of daily perceived mood during social interactions was adapted for social media.

##### Daily Negative Mood

To measure daily negative mood (not specific to social media use), participants answered a question on daily morning and evening surveys that asked, “How sad/down would you rate your mood today?” on a scale of 0 (not at all sad/down) to 100 (extremely sad/down).

#### Secondary Variables (for RQ3)

##### Demographics

Demographic information (e.g., age, sex, gender, sexual orientation, race, ethnicity) was captured via a standard demographic survey administered at the study baseline.

##### MacArthur Subjective Social Status Scale—Youth Version (Goodman et al., 2001)

The MacArthur Subjective Social Status Scale was used to measure socioeconomic status. In the youth version of this scale, children and adolescents are provided with a ladder with rungs numbered 1 (worst off) to 10 (best off). Participants rate their perceived status on the ladder indicating their status relative to those in society at large and one indicating their status relative to others at their school. Rating subjective social status in this way has been shown to be a better predictor of negative health outcomes relative to objective socioeconomic status both for adolescents (Goodman et al., 2001) and adults (Adler et al., 2000; Singh-Manoux et al., 2005).

##### Pubertal Development Scale (Petersen et al., 1988)

The Pubertal Development Scale, which has been shown to have high reliability and validity when measuring pubertal development among adolescents (Petersen et al., 1988), was used to measure pubertal development. Participants completed this measure according to their sex assigned at birth. The Pubertal Development Scale provides separate scores for biological females and males, where higher scores indicate more progress in pubertal development.

##### Mood and Feelings Questionnaire (Angold & Costello, 1987)

The Mood and Feelings Questionnaire was used to measure depressive symptoms. This scale has been shown to reliably measure depression among adolescents. In the current sample, Cronbach alpha was 0.95.

##### Multidimensional Anxiety Scale for Children (March et al., 1997)

The Multidimensional Anxiety Scale for Children was used to measure anxiety. This scale contains three subscales (physical symptoms, harm avoidance, and social anxiety). However, for the purposes of this study, we used the total score to measure overall anxiety. The Multidimensional Anxiety Scale for Children has been shown to have high validity and reliability among youth (March et al., 1997; Villabø et al., 2012; Wei et al., 2014). In the current sample, Cronbach alpha was 0.93.

##### Columbia Suicide Severity Rating Scale—Screening Version (Posner et al., 2011)

The screening (self-report) version of Columbia Suicide Severity Rating Scale was used to measure suicidal thoughts and behaviors. This scale asks participants to report on current and past experiences of suicidal ideation (including experiences of passive death wish and thoughts of killing oneself with or without a method, plan, or intent) and suicidal behaviors (e.g., attempts, aborted or interrupted attempts, preparatory behavior). Participants completed this measure at baseline and at the end of the study (after 1 month). This scale has been shown to have reliability and validity in measuring suicidal thoughts and behaviors among adolescents and adults (Posner et al., 2011).

### Data Processing and Cleaning

#### Processing

The smartphone sensing data were processed using the Reproducible Analysis Pipeline for Data Streams (Vega et al., 2021) in collaboration with the University of Pittsburgh’s Mobile Sensing + Health Institute (MoSHI). This open-source software processes the high-dimensional passive sensing data collected through AWARE and other platforms. The output of this processing pipeline was an intensive longitudinal dataset of meaningful features, binned into 1-h chunks across the study (i.e., each row represents 1 h of each day for each participant).

#### Data Cleaning

Following data processing, the data were cleaned in R (R Core Team, 2022) in order to combine the passive sensing data with the daily diary data, as well as baseline and follow-up data. The data were also cleaned to account for times when the AWARE app may not have been running properly within a given hour. Specifically, in some cases, AWARE captured 0.00 min of social media use within a given hour. Sometimes this represented no social media use or checking on behalf of the participant, but other times this was due to AWARE not running properly for most of that hour. We ultimately decided on a balanced approach to maximize data availability and integrity, based on prior research and our theoretical knowledge of adolescent phone usage (e.g., Low et al., 2021). We determined that, if no social media use data was captured in a given hour, AWARE had to be running for at least 50% of that hour to use that hour of participant data. In other words, if AWARE was running for less than 50% of a given hour and AWARE captured 0.00 min of social media use, that participant’s social media data would be “NA” for that hour. On the other hand, if AWARE was running properly for more than 50% of a given hour and AWARE still captured 0.00 min of social media use, the data would retain a value of 0.00 min of social media use and 0 social media checks.

Finally, for the purposes of day-level analyses (such as those in this study), hourly features were collapsed across the day level, similar to prior research (Rodman et al., 2021), where continuous measures (e.g., time spent on social media) were summed by day (12:00 a.m.–11:59 p.m.).

### Data Analytic Plan

Descriptive statistics were used to summarize demographic information (age, race, ethnicity, sex, gender, sexual orientation, and socioeconomic status), pubertal development, anxiety, depression, suicidal thoughts, attempts, and nonsuicidal self-injury. Key study variables (social media use and mood) were also summarized using descriptive statistics. Variability in key study variables was assessed using intraclass correlations.

Primary analyses (DSEM models) were conducted in Mplus (Muthén & Muthén, 1998). In order for models to converge, variables of interest needed to have some variability (i.e., not be the same number for each report). Thus, if participants did not report on a particular construct most days or reported the same value each day, models did not converge. Mplus defaults to an alpha threshold of *p* < 0.025 (as opposed to the more traditional *p* < 0.05). The more conservative *p* < 0.025 threshold was maintained for these analyses to minimize the likelihood of presenting spurious findings, especially given the exploratory nature of this project.

- RQ1 (Confirmatory): How are social media “screen time” and social media checking associated with positive and negative mood during social media use on a proximal scale, over time, for individual adolescents?

**Fig. 1:**
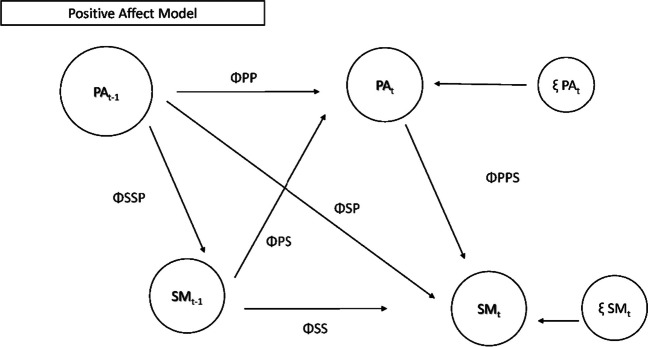
Model testing relationships between use patterns and positive mood during use. Note: PA = positive mood on SM; SM = social media use or checks; T-1 = prior timepoint; ΦPP = autoregressive parameter PA to PA; ΦSS = autoregressive parameter SM to SM; ΦPS = cross lagged parameter SM to PA; ΦSP = cross lagged parameter PA to SM; ΦSSP/PPS = correlation between PA and SM (same time point); ξ PAt = innovation variance PA; ξ SMt = innovation variance SM

**Fig. 2:**
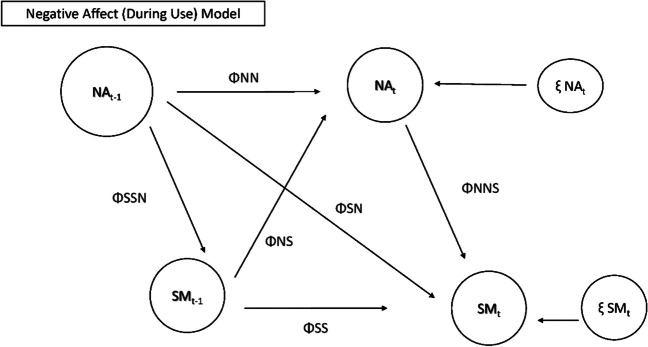
Model testing relationships between use patterns and negative mood during use. Note: NA = negative mood on SM; SM = social media use or checks; T-1 = prior timepoint; ΦNN = autoregressive parameter NA to NA; ΦSS = autoregressive parameter SM to SM; ΦNS = cross lagged parameter SM to NA; ΦSN = cross lagged parameter NA to SM; ΦSSN/NNS = correlation between NA and SM (same time point); ξ NAt = innovation variance NA; ξ SMt = innovation variance SM

To answer this question, we used DSEM to test our a priori models of the ways in which these variables are related. These models are based on prior exploratory work using multilevel modeling using the same sample (Dreier et al., 2024). DSEM is similar to traditional structural equation modeling but allows researchers to specify time-varying models/autoregressive models for repeated measures analyses. Figures 1 and 2 depict the models that were tested in RQ1 (positive and negative mood, respectively).

- RQ2 (Confirmatory): If and how do adolescents self-regulate their social media use (i.e., use or check it more or less) in relation to the general negative mood on the same day and following day?

**Fig. 3:**
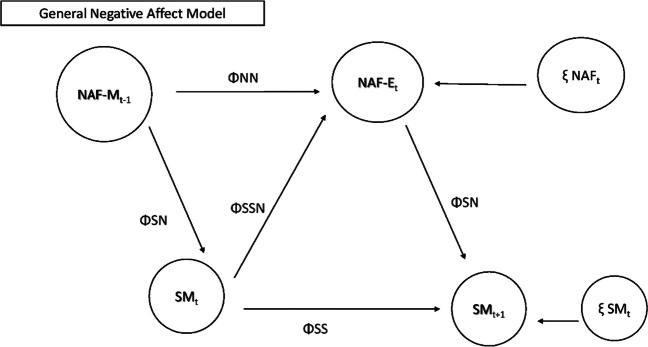
Models testing relationships between use and general negative mood. Note: NAF-M = morning negative mood; NAF-E = evening negative mood; SM = social media use or checks; T-1 = prior timepoint; T + 1 = next day; ΦNN = autoregressive parameter NAF to NAF; ΦSS = autoregressive parameter SM to SM; ΦSN = cross lagged parameter NAF to SM; ΦSSN = correlation between NAF and SM (same time point); ξ NAFt = innovation variance NAF; ξ SMt = innovation variance SM

To answer this question, we used DSEM to model patterns between these constructs. Figure 3 depicts the models tested for RQ2.

- RQ3 (Exploratory): What demographic and clinical characteristics are qualitatively associated with different social media use habits?

To answer this question, we qualitatively examined the results of RQ1 and 2 (i.e., individual patterns of mood while on social media, social media use, and daily mood). We then examined demographic (e.g., age, pubertal status, sex, gender, sexual orientation, race, socioeconomic status) and clinical (e.g., depressive symptoms, anxiety, suicidal thoughts and behaviors) information. Using this information, we present qualitative findings on ways in which different patterns of social media use may be related to mental health concerns (or lack thereof), developmental stage, and potential exposure to systemic/structural inequities (e.g., economic hardship, racism, sexism, homophobia).

## Results

### Sample Characteristics

Clinical characteristics, developmental, and demographic information are presented in aggregate in Table 1. Table 3 summarizes key study variables (social media use and mood). Table 4 presents bivariate correlations between key study variables.

**Table 3:** Descriptive statistics and intraclass correlations of core study variables

	*N*	*n*	*M*	SD	Range	Possible range	ICC
Daily measures							
Morning negative mood	19	358	23.04	28.18	0–100	0–100	0.45
Evening negative mood	18	358	20.92	26.07	0–100	0–100	0.5
Positive mood on social media	19	413	52.97	31.59	0–100	0–100	0.66
Negative mood on social media	18	298	14.9	20.13	0–94	0–100	0.34
Continuous measures (daily bins)							
Time spent on social media (minutes)	19	581	64.57	64.72	0–369.53	0–1440	0.16
Social media checks	19	581	134.58	138.87	0–1019	0–1440	0.24

*N* total number of participants, *n* number of observations, *M* mean (at daily level), *SD* standard deviation (at daily level), *ICC* intraclass correlations

**Table 4:** Bivariate correlations with confidence intervals for core study variables

Variable	1	2	3	4	5	6	7	8	9	10
1. Social media “screen time”										
2. Social media checking	.71**									
	[.39, .88]									
3. Morning negative affect	− .04	.05								
	[− .49, .42]	[− .41, .50]								
4. Evening negative affect	.06	.17	.90**							
	[− .40, .50]	[− .31, .58]	[.75, .96]							
5. Positive affect during social media use	− .38	− .11	− .22	− .11						
	[− .71, .09]	[− .53, .37]	[− .61, .26]	[− .54, .36]						
6. Negative affect during social media use	− .07	− .17	.44	.49*	.19					
	[− .52, .41]	[− .59, .32]	[− .03, .75]	[.02, .78]	[− .31, .60]					
7. MFQ	.35	.18	.39	.55*	− .33	.42				
	[− .12, .69]	[− .30, .58]	[− .07, .72]	[.13, .81]	[− .68, .15]	[− .06, .74]				
8. PDS	− .03	− .20	− .12	− .25	− .30	− .09	− .45			
	[− .48, .43]	[− .60, .28]	[− .55, .35]	[− .63, .23]	[− .66, .18]	[− .54, .39]	[− .75, .00]			
9. MASC	.42	.24	.31	.45	− .33	.39	.81**	− .32		
	[− .05, .73]	[− .24, .63]	[− .17, .67]	[− .01, .75]	[− .68, .15]	[− .10, .72]	[.55, .92]	[− .68, .16]		
10. SES—School	− .18	− .18	− .10	− .12	.16	− .00	− .19	− .04	− .13	
	[− .59, .30]	[− .59, .30]	[− .53, .37]	[− .54, .36]	[− .32, .57]	[− .47, .46]	[− .59, .29]	[− .48, .42]	[− .55, .34]	
11. SES—Society	− .36	− .40	− .16	− .25	.21	− .14	− .42	− .15	− .34	.64**
	[− .70, .11]	[− .72, .06]	[− .57, .32]	[− .63, .23]	[− .27, .61]	[− .57, .35]	[− .73, .04]	[− .56, .33]	[− .69, .13]	[.27, .85]

Values in square brackets indicate the 95% confidence interval for each correlation. The confidence interval is a plausible range of population correlations that could have caused the sample correlation (Cumming, 2014)

*MFQ* Mood and Feelings Questionnaire, *PDS* Pubertal Development Scale, *MASC* Multidimensional Anxiety Scale for Children, *SES—School* perceived socioeconomic status relative to others in one’s school, *SES—Society* perceived socioeconomic status relative to others in society

^*^p < .05

^**^p < .01

### Social Media Applications

A total of 645 phone applications were used by the 19 participants in this study. To isolate social media apps, three independent coders from the study team researched all phone applications used and rated whether each counted as social media. Following established definitions in the field (Carr & Hayes, 2015), applications were classified as social media if participants could create a profile and interact with others both synchronously and asynchronously for broad social networking purposes (which excludes narrower cases, like dating apps). Following this procedure, 28 applications were coded as social media apps, and data from usage of those apps were aggregated to create “social media” variables in the data set (including time spent on any of these apps in total and number of times any of these apps were checked). Refer to the “Data Processing and Cleaning” section for more information about the final dataset creation.

### Primary Analyses

Although the vast majority of the models (96 of 114) converged, 18 models did not due to missing data and/or no variance (e.g., participant reported the same number each day).

- RQ1: How are social media “screen time” and social media checking associated with positive and negative mood during social media use on a proximal scale, over time, for individual adolescents?

Table 5 summarizes patterns in social media “screen time,” social media checking, and positive and negative moods during use. The majority of adolescents (*n* = 15) did not self-regulate social media use “screen time” or checking in relation to their mood during social media use. Of those who did use social media in relation to mood during use (*n* = 4), four distinct patterns emerged. One adolescent (#3) had more social media screen time on days following a higher negative mood during use. Another adolescent (#4) felt a more positive mood on social media on days following more “screen time” and checked social media less on days following a higher negative mood during use. A third participant (#15) felt a higher negative mood during social media use on days when they used it more, but checked social media less the following day. The final participant (#16) checked social media more on days following when it elicited a stronger positive mood, but on days with more use, felt a less positive mood during use the following day.

- RQ2: If and how do adolescents self-regulate their social media use (i.e., use or check it more or less) in relation to the general negative mood on the same day and following day?

**Table 5:** Idiographic relationships between social media use, positive and negative mood during use, and general negative mood

	Same day		Next day		Next day (inverse relationship)	
#	*β*	CI	*β*	CI	*β*	CI
1						
NAF → SMU	0.051	[− 0.532, 0.533]	− 0.251	[− 0.590, 0.138]	–	–
NAF → SM Check	0.091	[− 0.433, 0.572]	0.049	[− 0.370, 0.449]	–	–
2						
NAF → SMU	0.445	[− 0.092, 0.768]	0.021	[− 0.007, 0.042]	–	–
NAF → SM Check	0.282	[− 0.234, 0.715]	**0.498**	**[0.076, 0.816]**	–	–
3						
PA → SMU	0.258	[− 0.137, 0.607]	0.083	[− 0.337, 0.482]	0.100	[− 0.341, 0.541]
PA → SM Check	0.316	[− 0.123, 0.670]	− 0.044	[− 0.465, 0.376]	− 0.067	[− 0.509, 0.407]
NA → SMU	0.335	[− 0.249, 0.844]	**1.970**	**[0.359, 3.314]**	0.000	[− 0.024, 0.023]
NA → SM Check	0.340	[− 0.259, 0.810]	0.241	[− 0.217, 0.677]	− 0.099	[− 0.580, 0.440]
4						
PA → SMU	0.327	[− 0.140, 0.737]	0.292	[− 0.104, 0.635]	**0.543**	**[0.151, 0.810]**
PA → SM Check	− 0.051	[− 0.480, 0.350]	− 0.035	[− 0.427, 0.371]	− 0.037	[− 0.484, 0.413]
NA → SMU	− 0.129	[− 0.600, 0.390]	− 0.022	[− 0.539, 0.518]	0.340	[− 0.205, 0.715]
NA → SM Check	− 0.333	[− 0.790, − 0.140]	** − 0.485**	**[− 0.931, − 0.005]**	− 0.100	[− 0.545, 0.371]
NAF → SMU	− 0.050	[− 0.310, 0.223]	0.344	[− 0.392, 1.101]	–	–
NAF → SM Check	0.086	[− 0.343, 0.491]	0.285	[− 0.147, 0.636]	–	–
5						
PA → SMU	0.294	[− 0.328, 0.824]	− 0.140	[− 0.648, 0.439]	0.211	[− 0.357, 0.711]
PA → SM Check	0.464	[− 0.116, 1.001]	− 0.123	[− 0.715, 0.475]	0.046	[− 0.556, 0.692]
NA → SMU	− 0.078	[− 0.638, 0.497]	− 0.154	[− 0.611, 0.360]	− 0.247	[− 0.712, 0.341]
NA → SM Check	− 0.242	[− 0.755, 0.314]	− 0.211	[− 0.715, 0.270]	− 0.218	[− 0.784, 0.392]
NAF → SMU	− 0.278	[− 0.666, 0.177]	− 0.008	[− 0.503, 0.513]	–	–
NAF → SM Check	− 0.355	[− 0.806, 0.089]	0.051	[− 0.474, 1.135]	–	–
6						
PA → SMU	0.004	[− 0.449, 0.476]	− 0.071	[− 0.501, 0.382]	− 0.194	[− 0.630, 0.302]
PA → SM Check	0.009	[− 0.455, 0.487]	− 0.066	[− 0.494, 0.404]	− 0.235	[− 0.660, 0.250]
NA → SMU	− 0.127	[− 1.250, 0.901]	− 0.238	[− 1.181, 0.623]	− 0.139	[− 0.462, 0.202]
NA → SM Check	0.052	[− 1.141, 1.001]	0.390	[− 1.017, 1.564]	− 0.062	[− 0.688, 0.685]
NAF → SMU	0.087	[− 0.350, 0.548]	− 0.021	[− 0.429, 0.459]	–	–
NAF → SM Check	0.090	[− 0.361, 0.548]	− 0.027	[− 0.469, 0.457]	–	–
7						
PA → SMU	0.204	[− 0.227, 0.609]	0.029	[− 0.413, 0.478]	0.029	[− 0.413, 0.478]
PA → SM Check	0.391	[− 0.092, 0.759]	− 0.061	[− 0.550, 0.443]	− 0.163	[− 0.542, 0.261]
8						
PA → SMU	− 0.024	[− 0.371, 0.351]	0.133	[− 0.238, 0.460]	0.113	[− 0.265, 0.468]
PA → SM Check	0.081	[− 0.302, 0.435]	0.114	[− 0.237, 0.446]	− 0.034	[− 0.396, 0.384]
NA → SMU	0.160	[− 0.234, 0.511]	0.174	[− 0.188, 0.506]	0.095	[− 0.286, 0.429]
NA → SM Check	0.290	[− 0.093, 0.614]	0.200	[− 0.182, 0.542]	0.039	[− 0.321, 0.382]
NAF → SMU	0.132	[− 0.224, 0.472]	0.001	[− 0.356, 0.335]	–	–
NAF → SM Check	0.163	[− 0.231, 0.496]	− 0.068	[− 0.391, 0.264]	–	–
9						
PA → SMU	0.394	[− 0.020, 0.742]	0.060	[− 0.336, 0.457]	0.255	[− 0.298, 0.821]
PA → SM Check	0.244	[− 0.149, 0.571]	0.163	[− 0.214, 0.536]	0.435	[− 0.029, 0.962]
NA → SMU	− 0.262	[− 1.021, 0.435]	− 0.143	[− 0.786, 0.554]	− 0.347	[− 0.791, 0.044]
NA → SM Check	− 0.128	[− 0.787, 0.330]	− 0.143	[− 0.912, 0.408]	− 0.104	[− 0.612, 0.285]
10						
PA → SMU	− 0.046	[− 0.395, 0.245]	0.100	[− 0.372, 0.506]	0.173	[− 0.271, 0.678]
PA → SM Check	− 0.030	[− 0.408, 0.313]	0.222	[− 0.330, 0.658]	0.106	[− 0.336, 0.557]
NAF → SMU	0.151	[− 0.558, 0.786]	0.086	[− 0.694, 0.728]	–	–
NAF → SM Check	0.202	[− 0.557, 0.794]	− 0.061	[− 0.796, 0.673]	–	–
11						
PA → SMU	0.294	[− 0.338, 0.850]	− 0.128	[− 0.721, 0.547]	0.481	[− 0.248, 0.989]
PA → SM Check	0.124	[− 0.456, 0.715]	− 0.260	[− 0.786, 0.423]	0.089	[− 0.582, 0.713]
12						
PA → SMU	0.031	[− 0.370, 0.433]	0.359	[− 0.049, 0.711]	0.250	[− 0.164, 0.624]
PA → SM Check	0.089	[− 0.373, 0.540]	0.258	[− 0.191, 0.629]	0.359	[− 0.070, 0.704]
NA → SMU	0.204	[− 0.223, 0.584]	− 0.050	[− 0.448, 0.353]	− 0.265	[− 0.636, 0.180]
NA → SM Check	0.143	[− 0.296, 0.515]	0.165	[− 0.281, 0.547]	− 0.172	[− 0.580, 0.281]
NAF → SMU	0.124	[− 0.253, 0.472]	− 0.164	[− 0.524, 0.265]	–	–
NAF → SM Check	0.113	[− 0.264, 0.520]	0.121	[− 0.280, 0.521]	–	–
13						
PA → SMU	0.559	[− 0.616, 1.392]	0.214	[− 0.594, 0.884]	0.532	[− 0.466, 1.070]
PA → SM Check	− 0.365	[− 0.868, 0.486]	− 0.515	[− 0.973, 0.604]	− 0.118	[− 0.758, 0.524]
NA → SMU	0.129	[− 0.635, 1.021]	− 0.310	[− 1.159, 0.561]	0.419	[− 0.451, 0.863]
NA → SM Check	− 0.034	[− 0.688, 0.646]	0.249	[− 0.768, 0.909]	− 0.134	[− 0.687, 0.484]
NAF → SMU	0.115	[− 0.212, 0.489]	0.135	[− 0.303, 0.539]	–	–
NAF → SM Check	0.077	[− 0.269, 0.421]	** − 0.406**	**[− 0.739, − 0.024]**	–	–
14						
PA → SMU	− 0.188	[− 0.991, 0.491]	0.428	[− 0.208, 1.312]	− 0.004	[− 0.319, 0.332]
PA → SM Check	0.392	[− 0.251, 1.216]	0.200	[− 0.425, 0.840]	0.298	[− 0.020, 0.700]
NA → SMU	0.056	[− 0.326, 0.441]	0.335	[− 0.102, 0.670]	0.060	[− 0.399, 0.496]
NA → SM Check	0.078	[0.363, 0.505]	0.219	[− 0.214, 0.595]	0.145	[− 0.353, 0.569]
NAF → SMU	0.250	[− 0.200, 0.587]	0.214	[− 0.245, 0.597]	–	–
NAF → SM Check	0.355	[− 0.090, 0.699]	0.326	[− 0.129, 0.702]	–	–
15						
PA → SMU	0.145	[− 0.175, 0.458]	− 0.123	[− 0.436, 0.223]	− 0.194	[− 0.583, 0.172]
PA → SM Check	0.317	[− 0.044, 0.611]	0.081	[− 0.267, 0.428]	0.080	[− 0.328, 0.480]
NA → SMU	**0.355**	**[0.013, 0.614]**	− 0.292	[− 0.598, 0.046]	0.179	[− 0.246, 0.620]
NA → SM Check	0.189	[− 0.162, 0.517]	** − 0.448**	**[− 0.708, − 0.103]**	0.247	[− 0.183, 0.619]
NAF → SMU	− 0.041	[− 0.413, 0.330]	0.160	[− 0.174, 0.457]	–	–
NAF → SM Check	0.167	[− 0.226, 0.484]	− 0.065	[− 0.418, 0.288]	–	–
16						
PA → SMU	− 0.421	[− 0.855, 0.129]	0.172	[− 0.191, 0.557]	** − 0.474**	**[− 0.753, − 0.151]**
PA → SM Check	− 0.065	[− 0.409, 0.289]	**0.398**	**[0.061, 0.688]**	− 0.008	[− 0.354, 0.357]
NA → SMU	0.774	[− 0.017, 1.443]	− 0.198	[− 0.965, 0.808]	− 0.074	[− 0.718, 0.636]
NA → SM Check	0.137	[− 0.490, 0.752]	− 0.159	[− 0.732, 0.426]	− 0.225	[− 0.702, 0.387]
NAF → SMU	0.237	[− 0.237, 0.630]	− 0.083	[− 0.483, 0.329]	–	–
NAF → SM Check	− 0.004	[− 0.331, 0.323]	− 0.145	[− 0.485, 0.236]	–	–
17						
PA → SMU	0.207	[− 0.638, 0.954]	0.096	[− 0.575, 0.831]	0.306	[− 0.612, 0.907]
PA → SM Check	0.141	[− 0.635, 0.895]	0.101	[− 0.519, 0.882]	− 0.024	[− 0.917, 0.769]
NA → SMU	0.279	[− 0.710, 1.278]	0.201	[− 0.407, 0.911]	0.693	[− 0.306, 1.053]
NA → SM Check	0.202	[− 0.825, 1.260]	0.283	[− 1.868, 2.739]	0.125	[− 0.907, 0.877]
NAF → SMU	0.049	[− 0.523, 0.669]	0.152	[− 0.490, 0.688]	–	–
NAF → SM Check	0.144	[− 0.576, 0.703]	0.025	[− 0.519, 0.639]	–	–
18						
PA → SMU	0.067	[− 0.816, 0.853]	− 0.233	[− 0.753, 0.564]	0.209	[− 0.384, 0.716]
PA → SM Check	0.354	[− 0.511, 0.903]	− 0.334	[− 0.792, 0.256]	− 0.148	[− 0.725, 0.635]
19						
PA → SMU	0.120	[− 0.291, 0.494]	− 0.391	[− 0.723, 0.009]	0.063	[− 0.383, 0.487]
PA → SM Check	0.248	[− 0.145, 0.585]	− 0.017	[− 0.037, 0.005]	0.000	[− 0.004, 0.004]
NAF→ SMU	− 0.253	[− 0.622, 0.214]	0.059	[− 0.381, 0.526]	–	–
NAF → SM Check	− 0.017	[− 0.544, 0.337]	− 0.368	[− 0.785, 0.065]	–	–

Models omitted are those that did not converge due to missing data. Bold denotes statistical significance (*p* < .025)

*β* standardized coefficient, *CI* 97.5% confidence interval, *SMU* social media “screen time,” *SM Check* social media checking frequency, *PA* positive mood during social media use, *NA* negative mood during social media use, *NAF* general negative mood (reporting in evening)

Table 5 summarizes patterns of social media “screen time” and checking in relation to general evening negative mood, while accounting for morning negative mood at the same-day and next-day levels. Most adolescents (*n* = 17) did not self-regulate their use in relation to general mood state. Two adolescents did, however, adjust their social media use in relation to their general mood state. One (#2) checked it more on days following a greater evening negative mood, whereas another (#13) checked it less on days following a greater evening negative mood.

- RQ3: What demographic and clinical characteristics are qualitatively associated with different social media use habits?

Table 6 summarizes demographic variables for each participant. Concrete patterns in social media regulation did not appear to emerge by age, race, sexual orientation, gender identity, pubertal status, or socioeconomic status, though these patterns may be interpreted in light of individuals’ demographic characteristics.

**Table 6:** Demographic characteristics by participant

#	Age	Sex	Gender	Race	Sexual orientation	SES—Society (1–10)	SES—School (1–10)	PDS (1–4)	MFQ (0–66)	MASC (0–117)	STB
1	16	Male	Boy	white	Heterosexual	7	7	3.667	0	20	None
2	16	Male	Nonbinary	white	Bisexual	4	5	2.5	50	85	SI, SA
3	16	Female	Girl	Black	Heterosexual	5	5	3.667	0	4	None
4	18	Male	Boy	white	Heterosexual	8	7	3.667	7	36	SI
5	16	Male	Boy	white	Heterosexual	8	6	2.833	15	49	SI, SA
6	16	Male	Boy	white	Bi-curious	6	8	3.5	7	56	None
7	15	Male	Boy	white	Heterosexual	7	9	3.5	2	39	None
8	16	Female	Girl	Black	Bisexual	4	5	3.667	12	42	None
9	15	Female	Girl	Black, white	Bisexual	7	5	3.333	9	46	None
10	14	Male	Boy	white	Heterosexual	7	7	3.333	6	20	None
11	16	Male	Boy	white	Heterosexual	7	5	3.1667	2	38	None
12	17	Male	Boy	white	Heterosexual	8	8	3.1667	6	36	None
13	15	Male	Boy	white	Heterosexual	5	4	3.5	8	52	SI, SA
14	17	Female	Girl	Black, white	Heterosexual	5	8	3.5	21	64	SI
15	16	Female	Girl	white	Heterosexual	6	6	3.83	10	42	None
16	16	Male	Boy	white	Heterosexual	9	10	2.83	4	39	None
17	16	Male	Girl	white	Queer	8	9	3.333	14	29	SI, NSSI
18	15	Male	Boy	white	Heterosexual	5	5	3	0	21	None
19	16	Female	Girl	white	Heterosexual	5	5	3.5	22	72	None

For both SES measures, 1 = much lower SES relative to these groups (society or school), and 10 = much higher SES relative to these groups. Ranges in headings indicate possible ranges for applicable measure

*SES—School* socioeconomic status relative to society at large, *SES—School* socioeconomic status relatives to others in the same school, *PDS* Pubertal Development Scale, *MFQ* Mood and Feelings Questionnaire (Depression), *MASC* Multidimensional Anxiety Scale for Children, *STB* suicidal thoughts and behaviors, *SI* suicidal ideation, *SA* suicide attempt(s), *NSSI* nonsuicidal self-injury

Although patterns did not emerge based on a history of suicidal thoughts and behaviors, anxiety, and depression, many individuals with a history of suicidal thoughts and behaviors did self-regulate their social media use in some way based on mood during or after use. One participant (#2) checked social media more on days following a higher negative mood. Another participant (#4) checked it more on days following higher negative mood during use, but then felt more positive during use on the days following more use. Finally, participant #13 used social media less on days following a stronger negative mood.

## Discussion

In this sample of 19 adolescents during the COVID-19 pandemic, most adolescents (68.4%) did not self-regulate their social media use in relation to how they felt during use or in general, at the same-day or next-day levels. In other words, most adolescents did not appear to be attending to their mood during or after use in deciding how long to spend on social media or how frequently to check social media. The fact that most adolescents did not *downregulate* use when they felt more negative mood during use is notable. It is possible that many adolescents may not be aware of the moment when social media elicits a negative mood and do not seem to adjust their behavior on the same day or next day when social media brings up negative feelings. That said, a minority (*n* = 6) of adolescents did self-regulate their social media use based on their mood during (*n* = 4) or after use (*n* = 2). Although these findings are preliminary and may be the product of many individual nuances, they provide templates for understanding how social media use guidelines may be well-tailored for different adolescents, based on individual characteristics (e.g., mental health history and other demographic characteristics).

Consider the cases of participant #15 versus participant #2. At first, it may appear that participant #15 has an “adaptive/harmful” pattern of use whereas participant #2 has a “maladaptive/helpful” pattern of use. Participant #15 tends to feel more negative mood during social media use on days when she uses it more, but then seems to learn from this, such that the next day, she checks social media less. On the other hand, participant #2 checks social media more frequently on days following a stronger general negative mood. If social media is conceptualized as a “negative” or “maladaptive” in and of itself, then participant #2’s pattern would be maladaptive. However, participant #2 also has several important characteristics to consider: they identify as nonbinary, bisexual, and have a history of suicidal thoughts and attempts. Social media can be an important mental health protective factor for LGBTQIA + adolescents—offering social connection and access to identity-related support that may not be available in person (Craig et al., 2021; Escobar-Viera et al., 2022). Given these data were collected during the COVID-19 pandemic, when adolescents were mostly home with their families, social media may have represented an especially important way for adolescents to connect, particularly for LGBTQIA + adolescents who have disclosed their identity to parents or other family members (Fish et al., 2020). Social media use can also be protective for adolescents with recent suicide attempts—likely facilitating important social support as well (Hamilton et al., 2021a, 2021b, 2021c). Given this, for participant #2, checking social media *more* following a higher negative mood could represent an adaptive coping skill.

Importantly, these interpretations about individual adolescents’ social media use behaviors are highly speculative. These data do not have information on whether LGBTQIA + adolescents in our sample were “out” to their parents or on what exactly they were doing on social media. Future research should work to fill these gaps so researchers may better understand how adolescents’ identities and characteristics interact with unique social media use patterns to produce different mental health outcomes (Charmaraman et al., 2022a). Guidelines could therefore also be crafted with nuance—centering considerations for individual adolescents, while still using scientific research as a basis.

### Looking Ahead: Applying These Findings to Emerging Research

Research consensus has been building that adolescent social media use may be associated with negative mental health outcomes for some adolescents, likely when they use social media in particularly harmful or “problematic” ways (Beyens et al., 2020; Montag et al., 2024; Valkenburg et al., 2022). Yet, researchers have yet to develop a concrete definition of what “harmful/problematic” versus “helpful” social media use looks like (Montag et al., 2024). Results from this study do not yet provide concrete, steadfast guidelines on how adolescents should use social media in order to promote mental health and minimize harm. Nonetheless, findings may begin to build a template for understanding how social media may be helpful or harmful for different adolescents.

Future research should investigate strategies that may increase helpful social media use and decrease harmful use among adolescents. Emerging evidence demonstrates that parental rules around social media do not effectively buffer against mental health outcomes, whereas open communication with parents about social media use does buffer against this relationship (Barry & Kim, 2024). This suggests that parents and other relevant stakeholders should engage in open dialogue with teenagers to help them use social media in the healthiest way possible for them. Future research should continue to probe for effective strategies to help adolescents maximize the benefits and minimize harms of social media use.

### Limitations

This study should also be interpreted in light of several important limitations. First, the overall sample size (19 adolescents) was relatively small. Additionally, the number of observations for each participant (roughly 30 days of reports) was sufficient for our models but still may not have been powered to detect smaller effects. Second, this sample was mostly white, entirely non-Hispanic/Latine, and most participants were heterosexual and cisgender. This limits the generalizability of these findings, especially given the importance of considering identity in relation to each adolescent’s unique patterns. Third, given the passive smartphone sensing software (AWARE) is unable to access applications-related sensors on iOS platforms, participants needed to use Android phones to take part in the aspects of the study included in this project’s analyses. That said, given Android phones are sold at a broader price point than iPhones, this contributes to considerable socioeconomic diversity in this sample, which strengthens the generalizability of these results. Fourth, although daily diary surveys partially mitigate recall bias, questions about mood during social media use were asked only once per day. There may have been some recall bias for participants when reflecting on experiences from earlier in the day. Additionally, although daily measures about mood were adapted based on existing gold-standard methods of asking about mood (the VAS), these questions themselves were not yet from validated measures. Fifth, in order to examine the effects of social media at the daily level, social media use and checking were summed by day (12:00 a.m.–11:59 p.m.), which imposes some researcher judgment on these data and may wash away some individual variability. Sixth, suicidal thoughts and behaviors were measured using a self-report measure, the Columbia Suicide Severity Rating Scale—Screening Version, which is a validated measure of these constructs (Posner et al., 2011). However, given the sensitive nature of these constructs, it is possible that adolescents were not as forthcoming as they may have been in a live clinical interview. Lastly, these data were collected in 2020 during the COVID-19 pandemic and were collected over a relatively short period of time (April-November 2020). This may influence the results of this study and limit generalizability to more recent timeframes, given that adolescents used different social media platforms during this time (e.g., TikTok became much more popular) and may have been using their phones more without in-person socialization. That said, emerging research indicates that, although these changes did occur at the start of the COVID-19 pandemic, many of those changes have taken hold and remain true of adolescents’ patterns of use (Anderson et al., 2023).

That said, this study has several notable strengths, including the use of both passive smartphone sensing and experience sampling (daily diary) methods to measure adolescent social media use. The use of dynamic structural equation modeling, an advanced statistical technique, also allowed for nuanced conclusions about the ways in which individual adolescents’ social media use and mood were associated at a daily level. Finally, this study is the first to provide a template for considering the ways in which adolescents’ individual characteristics may be centered within public health and clinical recommendations for adolescent social media use.

## Conclusions

Overall, this project provides preliminary insight into how different adolescents self-regulate (or do not self-regulate) their social media use in relation to their mood. Findings highlight that most adolescents are not self-regulating social media use, indicating that interventions focused on teaching adolescents to recognize how they feel during use could be an effective approach. Additionally, nuance in different use patterns for adolescents with different identities and mental health characteristics highlights the importance of considering these factors when crafting recommendations. Person-specific/idiographic approaches are a critical next step in understanding the unique ways in which social media use and mental health outcomes may be associated with different adolescents.

## Data Availability

Data are available upon request.
